# Analysis of Offensive Performance in 5-A-Side Soccer for the Blind: An Exploratory Study

**DOI:** 10.3390/sports14070312

**Published:** 2026-07-22

**Authors:** Boryi A. Becerra-Patiño, Juan David Paucar-Uribe, Rodrigo Yáñez-Sepúlveda, José Francisco López-Gil, José Pino-Ortega

**Affiliations:** 1 Programa de Doctorado en Ciencias de la Actividad Física y del Deporte, University of Murcia, San Javier, 30720 Murcia, Spain; boryialexander.becerrap@um.es; 2 Faculty of Physical Education, National Pedagogical University, Bogota 480100, Colombia; jdpaucaru@upn.edu.co; 3 Faculty of Education and Humanities, School of Sport Sciences, Universidad Andres Bello, Viña del Mar 2520000, Chile; rodrigo.yanez.s@unab.cl; 4 Department of Sport Sciences, Faculty of Sport and Health Sciences, Fit Generation Research Institute, AD500 Andorra la Vella, Andorra; 5 School of Medicine, Universidad Espíritu Santo, Samborondón 092301, Ecuador; 6 Vicerrectoría de Investigación y Postgrado, Universidad de Los Lagos, Osorno 5290000, Chile; 7 Faculty of Sport Science, University of Murcia, 30100 Murcia, Spain; josepinoortega@um.es

**Keywords:** visual impairment, athletic performance, sports tactics, blind football, match analysis, spatiotemporal metrics

## Abstract

**Background**. Five-a-side soccer for the blind (F5) is a Paralympic sport that has been the subject of research aimed at identifying variables that influence performance. However, despite its growing popularity, there is limited scientific literature on quantitative analyses of offensive performance in this sport. **Objective**. To analyze 164 offensive plays by the Argentine men’s national team during the 2022 International Blind Sports Federation (IBSA) Copa América to determine the spatiotemporal progression and the decline in intra-match offensive performance. **Materials and Methods**. Each play was reconstructed with its progression vector, its finishing zone, and its absolute game minute. Multivariate logistic regression models and weighted trend analysis (Cochran–Armitage) were applied to examine the relationship between these variables and the offensive outcome (shot on-target and goal). **Results**. Plays originating from high-pressure recovery showed a favorable trend toward shooting on-target. The goal rate showed an empirical decline from 26.1% in the 6th–10th minutes to 6.7% in the final minutes, without reaching statistical significance. **Conclusions**. The results of this study suggest that the magnitude of the offensive progression vector and offensive effectiveness show an asymmetry toward the right side, as well as a pattern consistent with an intra-match decline toward the end of games. These variables appear to influence the offensive performance of the Argentine men’s national blind football (F5) in the 2022 Copa América. These findings provide one of the first integrated vector analyses of offensive performance in F5 and propose metrics applicable to coaching staff, the design of specific training programs, and the functional assessment of athletes with visual impairments; therefore, further studies are needed to analyze these metrics in different official competitions and to account for various competitive levels. It is recommended to design training exercises focused on recovery under high pressure near the right side of the offensive zone and maintain the intensity of play in the final minutes of the game.

## 1. Introduction

Five-a-side soccer for the blind (F5) has been a Paralympic sport since the 2004 Athens Games and is governed by the International Blind Sports Federation (IBSA) [1]. The game is played by four field players who are totally or nearly totally blind and have no light perception; they wear eye masks to ensure competitive equality. The sport is played on a 40 × 20 m rectangular field surrounded by side zones (kickboards) that keep the ball in play most of the time [2]. This leads totally blind players (B1 classification) to rely on their spatial orientation as one of their primary skills [3].

There are three regulatory requirements that make it possible to play the sport. First, the ball contains internal bells that produce a continuous sound as it moves, allowing players to locate it by sound [4]. Second, regulations require the audience to remain silent during active phases of play to preserve the acoustic field [5]. Third, each team has three auditory guides: the goalkeeper, who may be sighted; the coach, positioned on the sideline; and a tactical assistant located behind the opposing goal to help players use acoustic signals to identify their position on the field, distance to the goal, and scoring opportunities [6].

As a result, postural balance [7] and playing position emerge as key determinants in F5, since the latter influences the execution of offensive actions in team sports [8,9]. Likewise, players’ kinematic profiles are influenced by sports actions related to passing and shooting [10,11]. This poses a challenge for players as they must adapt to a sustained cognitive load through spatial orientation without visual information [12,13] originating from the ball, teammates, opponents, and coaches [2]. The integration of all this information into quantitative performance estimates, however, has received very limited attention.

Some studies have reported that the effectiveness of shots on goal [14] and the characterization of specific technical–tactical actions in the game, such as ball control, dribbling past an opponent, and shooting, are key determinants for F5 players [15,16]. Other studies have sought to identify differences between official tournaments by analyzing tournament phases, timing of shots, shot outcomes, game status, starting zone, and type of progression [16]. Most analyses of offensive tactical performance have been conducted using video analysis of official competitions [17], particularly through the observational instrument for evaluating goal-scoring attempts in F5 for the blind (IOLF5C), as validated by Gamonales et al. [18]. However, although there are some studies analyzing shots on goal in world championships [16], no studies have been identified to date that examine spatiotemporal progression and the decline in intra-match offensive performance in F5.

A systematic review of F5 research that analyzed offensive performance in F5 has determined that shooting accuracy in offensive zones with minimal opposition is the most influential variable; however, scientific output in this discipline remains limited, so further studies are needed to better understand tactical performance in F5 [19,20]. In this regard, knowing which variables are most studied and which are least explored could help provide a clearer picture of performance in F5, thereby enabling the modeling of behavior aligned with the sport’s objectives [21].

Another gap lies around in-game performance decline. While some studies have described declines in external variables toward the end of matches in soccer and futsal [22,23] there are limited studies that have examined this phenomenon in adapted sports, particularly in F5. This gap is particularly relevant in F5, where fatigue affects not only technical precision and tactical actions, but especially decision-making speed, an essential factor in performance in team sports [24,25] but also dynamic auditory processing capacity, as it is an essential component for locating the ball, teammates, and opponents.

Therefore, in statistical soccer analysis, the progression vector measures the magnitude and direction of the ball’s movement toward the opposing goal variables of great interest in the analysis of team sports such as F5, as they help filter out the noise from simple pass counts to identify the plays that actually advance the ball into dangerous zones such as the attacking third and the penalty area. With this in mind, this framework aims to analyze how offensive behavior changes over the course of the game, making it possible to identify patterns of change rather than just average values.

Due to the lack of scientific literature analyzing indicators of offensive tactical performance, there is a need to expand the body of knowledge in F5. Consequently, the objective of this study was to analyze offensive actions to determine the spatiotemporal progression and the decline in intra-match offensive performance of the Argentine men’s national team during the 2022 IBSA Copa América.

## 2. Materials and Methods

### 2.1. Research Design

A retrospective observational study [26] was conducted during the IBSA Blind Football Copa América held in Argentina. Data were collected using a scoring system based on six matches. The study included the first-round matches against the national teams of Peru (Match 1), Brazil (Match 2), Mexico (Match 3), and Chile (Match 4), as well as the final-round matches against Colombia (Match 5) and Brazil (Match 6).

### 2.2. Participants

Eight field players from the Argentine national team who competed in the tournament under IBSA regulations were analyzed. Under these regulations, four field players with a B1 classification compete, along with a goalkeeper who may be fully sighted [27]. The inclusion criterion for the analysis was that each field player must have participated in at least one recorded action. This study utilized fully anonymized data from official IBSA YouTube broadcasts and public tournament records. Individual players’ names were replaced with numerical codes prior to analysis. Furthermore, the various principles established in the Declaration of Helsinki [28] regarding privacy and data handling were adhered to, particularly given that the analysis involved athletes with disabilities.

### 2.3. Actions Analyzed

For each match, all recorded attacking plays by the Argentine national team were analyzed (*n* = 164). Sixteen plays lacked recorded origin coordinates and were excluded from the logistic model; all sixteen were goals (14 from open play or set pieces and 2 penalties), distributed across opponents roughly in proportion to the team’s scoring output (Peru = 6, Colombia = 5, Mexico = 3, Chile = 2; none against Brazil, which conceded no goals). Because the missing coordinates affected goals exclusively, this exclusion was informative rather than random with respect to the outcome: prevalence fell from 42.1% (69/164) to 35.8% (53/148) for shot on-target and from 17.1% (28/164) to 8.1% (12/148) for goals. The multivariable estimates therefore describe the predictors of a shot on-target among the 148 plays with a reconstructable origin vector and are conditional on that subset (see Table 4 and Figure 5).

### 2.4. Variable Coding

Each action was described by fifteen variables: (1) identifier; (2) date; (3) opponent; (4) match number; (5) tactical category; (6) minute of play; (7) origin coordinates (Xi, Yi) in the field reference system; (8) end coordinates (X, Y) in meters; (9) leg used (right, left, or body); (10) type of combination (individual or collective); (11) origin of the play (open play, high-pressure recovery, set piece, counterattack, or penalty); (12) half (first or second); (13) outcome (goal, saved, missed, out of bounds, blocked, post); (14) player taking the shot; (15) additional incidents. The playing area was defined according to IBSA regulation dimensions (40 × 20 m, surrounded by kickboards). The coordinate system was established with its origin at the opponent’s goal line and the X-axis extending toward the center of the court. The recording of actions and the reconstruction of coordinates were performed through systematic observation of the tournament’s official video broadcasts. In the present study, the inter- and intra-observer reliability of the scoring system was not formally quantified (e.g., Cohen’s Kappa, intraclass correlation coefficient, or standard error of measurement on a recoded subsample), which is explicitly acknowledged as a limitation (see limitations and future research).

### 2.5. Derived Variables

The following indicators were calculated using the coordinates of the starting and ending points: (1) magnitude of the progression vector, defined as the Euclidean distance between the two points; (2) distance to the goal at the moment of completion, defined as the Euclidean distance between the finishing point and the center of the goal. The absolute minute of play was calculated by adding 20 min to the second-half actions, and five-minute intervals and quartiles were constructed for trend analysis.

### 2.6. Outcome Variables

Two binary outcomes were defined. The primary outcome was a shot on-target, a category that grouped the outcomes’ “Goal” and “Saved,” while the secondary outcome was a goal. Any outcome that did not result in a shot on-target was considered a blocked or missed attempt. The secondary outcome (goal) was not modeled using multivariate logistic regression due to insufficient events: of the 28 plays that resulted in a goal, only 12 had reconstructable origin coordinates; the remaining 16 goals were excluded due to the lack of an origin vector (see actions analyzed) so that the modelable subset contained only 12 events compared to the model’s 8 parameters, well below the recommended threshold of events per variable. Therefore, goals were analyzed only descriptively (by opponent, zone, origin, and half of the match) and in the intra-match decay analysis, reserving multivariate modeling for the primary outcome. In sum, the shot on-target is the study’s primary (confirmatory) outcome and the only outcome submitted to multivariable modeling, whereas the goal is treated throughout as a secondary, exploratory and purely descriptive outcome.

### 2.7. Statistical Analysis

Each continuous variable was described using the mean and standard deviation, while categorical variables were described using frequency and proportion. The association between offensive variables and the outcome was examined using multivariate logistic regression with the primary outcome as the dependent variable. Continuous predictors (magnitude of progression, distance to the goal, and minute of play) were standardized beforehand. Categorical variables were modeled using dummy variables. The two recorded penalties were excluded from the model; in addition to lacking origin coordinates, they constitute a set-piece play without comparable spatial progression, so the “penalty” category of the play origin variable was not represented among the predictors in the logistic model. The quality of the fit was evaluated using the Akaike Information Criterion (AIC), Cox–Snell pseudo-R^2^, and Nagelkerke pseudo-R^2^ [29,30], as the latter allows for correction of the upper bound of the Cox–Snell pseudo-R^2^ when the prevalence of the outcome is moderate. In interpretive terms, these pseudo-R^2^ statistics express the share of outcome variability accounted for by the model relative to a null (intercept-only) model, with higher values denoting greater explanatory capacity; unlike the R^2^ of linear regression they seldom approach 1 in behavioral data, so values in the 0.05–0.10 range indicate a weak-to-modest fit. The adequacy of the model was further verified with a standard battery of regression diagnostics: multicollinearity was screened with variance inflation factors (VIF) and pairwise predictor correlations; the linearity of the continuous predictors on the logit scale was tested with the Box–Tidwell procedure; influential observations were examined through Cook’s distance, leverage (hat) values and standardized residuals, refitting the model after excluding flagged cases to confirm the stability of the estimates; and calibration was assessed with the Hosmer–Lemeshow goodness-of-fit test, the calibration slope, and the Brier score.

The decline in in-game performance was examined using three complementary approaches: (1) quadratic logistic regression with absolute minute as the sole predictor; (2) chi-square test and Cochran–Armitage linear trend test on the match quartile × shot on-target contingency table; and (3) visual inspection of the empirical means by five-minute intervals along with their confidence intervals. Heterogeneity among opponents in the magnitude of the progression vector was tested using one-way analysis of variance (ANOVA) and the nonparametric Kruskal–Wallis test. A two-tailed *p*-value < 0.05 was considered statistically significant. All analyses were performed using Python 3.11 [31] and SciPy 1.11, statsmodels 0.14, pandas 2.1, matplotlib 3.8, and NumPy [32]. Additionally, the available statistical power for the primary predictor (magnitude of the progression vector) was estimated using Monte Carlo simulation (3,000 replicates), assuming an Odds ratio (OR) of 0.68 per standard deviation and the prevalence of the outcome observed in the modeled subset (35.8%; 53/148), and this was compared with the analytical approximation for logistic regression proposed by Hsieh et al. [33]. Monte Carlo simulation was preferred for the power analysis because the closed-form approximations for logistic regression rely on large-sample assumptions that are unreliable at this sample size and outcome prevalence; the simulation instead repeatedly regenerates datasets under the observed design and records the proportion of replicates in which the primary effect is detected.

## 3. Results

### 3.1. Characterization of the Corpus of Plays

A summary of the 164 offensive plays analyzed is presented in Table 1. 17.1% (*n* = 28) resulted in a goal, and 42.1% (*n* = 69) resulted in a shot on-target. The plays were concentrated in the finishing zone near the goal (X = 8.4 ± 5.2 m), with marked lateral dispersion (Y = 8.9 ± 4.1 m, asymmetry toward the right side of the field). Table 1 also shows the descriptive statistics (mean and standard deviation) for the number of attacks (*n*), goals, shots on-target, conversion and on-target percentages, magnitude of the progression vector, and final distance to the goal by an opponent. The data for Brazil include both group stage matches and the final.

**Figure 1:**
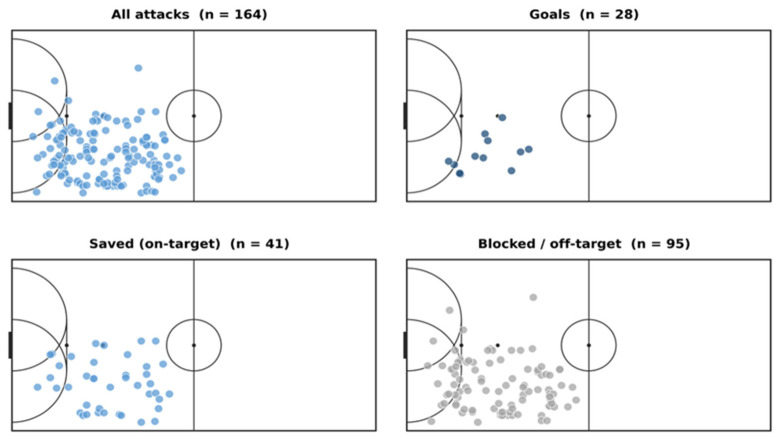
Spatial distribution of the final points of attacking plays. The figure shows the end points of *n* = 164 attacking plays on a half-field (40 × 20 m, according to International Blind Sports Federation (IBSA) blind football regulations) by outcome category. Coordinates follow the IBSA reference system, with the origin at the attacked goal line and the X-axis pointing toward midfield; goals, saved shots, and off-target or blocked attempts are distinguished by marker color. *n*: number; m: meters.

Figure 1 shows the spatial pattern of finishing attempts broken down by outcome: goals were mostly concentrated in a lateral zone near the goal, while blocked attempts or those outside the goal were scattered throughout the attacking zone.

### 3.2. Variability Among Opponents

Offensive effectiveness varied significantly depending on the opponent, as shown in Table 1. It is worth noting that, against Peru and Colombia, the Argentine team achieved conversion rates of 24.4% and 22.2%, respectively, while against Brazil the strongest opponent in the sport and the reigning world champion no goals were scored despite 24 offensive plays recorded in the two matches (group stage and final phase), with only 12.5% of shots on-target. Meanwhile, the mean magnitude of the progression vector shows statistically significant differences among the competitors (ANOVA: F = 3.06; *p* = 0.019; eta-squared (η^2^) = 0.079; Kruskal–Wallis: H = 11.77; *p* = 0.019; both tests on plays with progression vectors, *n* = 148).

**Figure 2:**
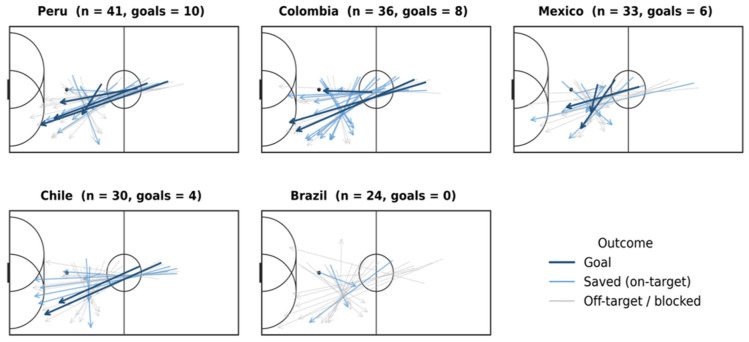
Attacking progression vectors by opponent. Each arrow runs from the reconstructed origin of an attack to its finishing point; color and thickness encode the outcome category (goal, shot on target, off-target/blocked), and panels are shown per opponent.

There is heterogeneity against Brazil, where attack vectors dispersed without converging toward the goal, in contrast to the clearly directional pattern observed against Peru and Colombia. This may be due to the ability to reduce the opponent’s effective offensive playing space. Thus, Figure 2 depicts the progression vectors of attacks by opponent. Each arrow connects the origin of an attack (tail) with its endpoint (head). The color and thickness of the vector indicate the outcome category. The graph for the Argentina-Brazil match illustrates the lack of convergence toward the goal observed in both matches.

### 3.3. Multivariate Predictors of Shots On-Target

The multivariate logistic regression model (*n* = 148 plays; AIC = 201.4; Nagelkerke R^2^ = 0.070) shows that the magnitude of the forward vector was inversely related to the probability of a shot on-target, after simultaneously adjusting for origin, combination, distance from the goal, and minute of play (β = −0.387; OR = 0.68; 95% CI: 0.46–1.00; *p* = 0.051). Table 2 shows the estimates of the coefficients, standard errors, odds ratios with 95% confidence intervals, and *p*-values from the final logistic regression model for the on-target outcome.

**Figure 3:**
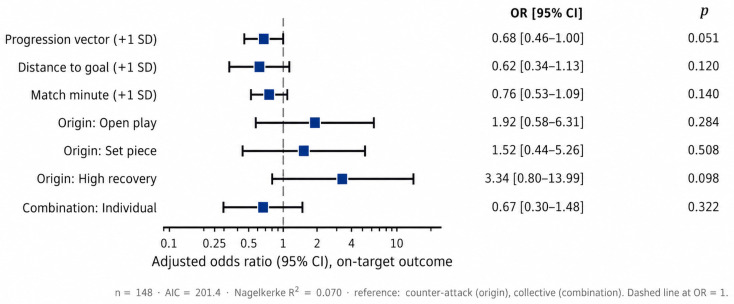
Multivariate predictors of on-target attacks. Forest plot of adjusted odds ratios (OR) as markers with 95% confidence intervals (lines) on a logarithmic scale from the multivariable model (*n* = 148); reference categories are counter-attack (origin) and collective play (combination), and the vertical line marks OR = 1. *n*: number; %: percentage.

Plays originating from high-pressure recovery showed a trend though not statistically significant toward greater odds of an on-target shot relative to counter-attacks (OR = 3.34; 95% CI: 0.80–13.99; *p* = 0.098). The remaining covariates such as open play, set pieces, individual combinations, distance from the goal, and minute of the match did not show statistically significant associations, although their coefficients followed the expected direction in all cases. Figure 3 depicts the multivariate logistic regression predictors of successful attacks based on the forest plot of odds ratios (markers) with 95% confidence intervals (lines) on a logarithmic scale, while the reference levels are shown as counterattack (origin) and collective play (combination).

The magnitude of the progression vector was the only predictor that approached significance (*p* = 0.051). Overall, given the study’s limited power, these estimates are better interpreted through their effect sizes and confidence intervals than through dichotomous significance: the progression-vector effect (OR = 0.68 per +1 SD; 95% CI 0.46–1.00) and the high-pressure recovery effect (OR = 3.34; 95% CI 0.80–13.99) are of practically meaningful magnitude, yet their intervals include values close to the null and must be regarded as exploratory.

Model diagnostics supported the adequacy of the fit. No problematic multicollinearity was present (all VIF < 3.0; the two spatial predictors progression vector and distance to goal showed the highest values, 2.9 and 2.8, consistent with their moderate correlation, r = −0.78). The Box–Tidwell test confirmed linearity on the logit scale for the three continuous predictors (all *p* > 0.60). No observation was unduly influential: the maximum Cook’s distance was 0.03, far below the conventional threshold of 1, no leverage value exceeded 2p/n, and refitting the model after excluding the most influential points left every coefficient essentially unchanged in direction, magnitude, and significance. The model was adequately calibrated (Hosmer–Lemeshow χ^2^ = 4.02; df = 8; *p* = 0.86; calibration slope ≈ 1.0; Brier score = 0.22), with an apparent area under the receiver operating characteristic (ROC) curve of 0.63, in line with the modest Nagelkerke R^2^ and reinforcing the interpretation of a weak but well-behaved model.

### 3.4. Intra-Match Decline in Offensive Performance

Table 3 summarizes the counts, outcome rates, and progression vector, broken down by half of the match. It confirms that there is a higher prevalence of goals and shots on-target in the first half.

Meanwhile, the empirical probability of scoring a goal showed a pattern consistent with a gradual decline. After an initial value of 14.3% in the first five minutes, the rate peaked at 26.1% between the 6th and 10th minutes and trended downward to 6.7% between the 36th and 40th minutes of the match. The probability of a shot on-target followed a similar pattern, with values close to 50% in the first half and a reduction to 33% in the final phase. However, neither the chi-square test by match quartile (χ^2^ = 0.78; *p* = 0.676) nor the Cochran–Armitage linear trend test (Z = −0.85; *p = 0.395 for shots on-target*; Z = −1.18; *p* = 0.236 for goals) reached statistical significance. In effect-size terms this represents a relative reduction of ≈74% in the goal rate between its early-match peak (minutes 6–10) and the closing minutes; the wide uncertainty at this sample size, however, means the temporal decline should be read as a descriptive trend rather than an established effect.

**Figure 4:**
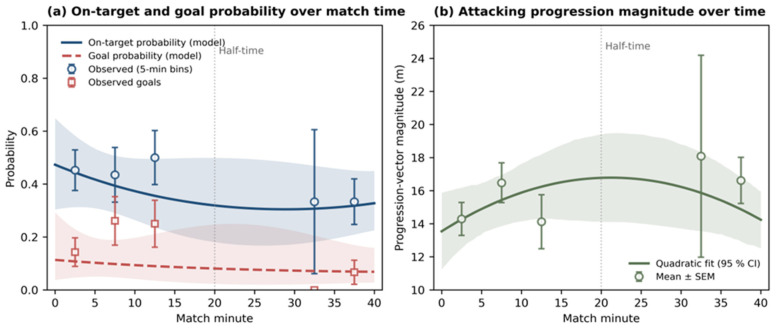
Intra-match performance trajectories. (**a**) Empirical and modeled probabilities of shots on-target (blue) and goals (red) over the course of the match; quadratic logistic regression fits with 95% confidence intervals. (**b**) standard error of the mean (SEM) of the progression vector over the course of the match, with quadratic fit and 95% bootstrap confidence interval. The dotted vertical line indicates halftime. The figure shows the temporal evolution of offensive performance during a blind soccer match (40 min of play). For (**a**): On-target and goal probability over match time, where the solid blue line represents the model’s estimated probability that an attack will end with a shot on-target; and Panel (**b**): Attacking progression magnitude over time, where the green line represents the quadratic model describing the magnitude of offensive progression (meters traveled toward the opponent’s goal). %: percentage.

The quadratic term of the decay model was also not significant (β = 0.0009; *p* = 0.403), although the fitted curve described a trough near the 28th minute, recovering slightly toward the end of the match (Figure 4a). The magnitude of the progression vector exhibited a curvilinear pattern, increasing from 14.3 m in the initial phase to a peak of 16.8 m around the 22nd minute, then declining to 14.3 m toward the end (Figure 4b). Meanwhile, Figure 4b shows the magnitude of offensive progression (m) over the course of the game. This variable represents the distance or advances a team makes toward the opponent’s goal during its offensive actions, noting that offensive progression increases from the first minutes of the match until reaching its peak around the 20–25 min mark (near halftime) and subsequently, the magnitude gradually decreases during the second half.

### 3.5. Spatial Effectiveness by Zone

Table 4 shows the count of attacks, goals, shots on-target, and conversion rates by combination of depth and lateral zone of the field. Zones with fewer than three attacks are excluded. The two plays in the left-wing zone (*n* = 2 in total) are excluded from the table but included in all descriptive totals; the sum of *n* across all zones shown is 146.

**Figure 5:**
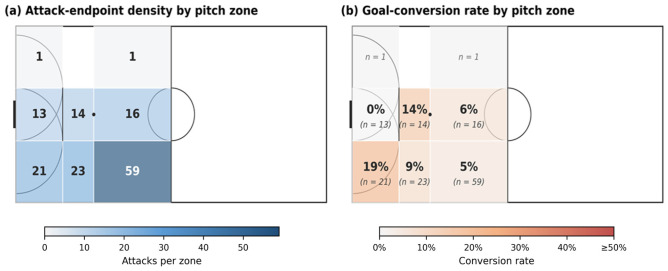
Spatial efficacy of attacking actions. (**a**) Density of completed attacking actions by zone of the field, showing a lateral concentration on the right side. (**b**) Percentage of goals by zone of the field, expressed as a percentage of attacks that result in a goal; zones with fewer than three attempts are shown without an estimate.

The distribution of terminations was markedly asymmetrical (Figure 5a). Thirty-six percent of the plays (*n* = 59) culminated in the deep right flank (X = 10–20 m, Y = 0–7 m), compared with only 2 plays on the corresponding left flank. Effectiveness was also heterogeneous (Figure 5b). In turn, the area near the goal on the right flank (0–6 m, 0–7 m) had the highest conversion rate (19.0%), followed by the central intermediate zone (6–10 m, center, 14.3%). The more distant zones showed significantly lower conversion rates (5–6%).

### 3.6. Origin and Type of Combination

**Figure 6:**
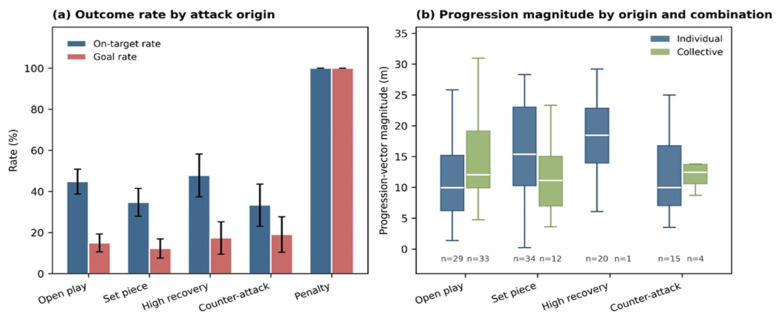
Tactical context of attacking play. Panel (**a**) shows on-target and goal rates by play origin; panel (**b**) shows progression-vector magnitude by combination type (individual vs. collective), summarizing the 164 recorded attacking plays. vs: versus.

The distribution by play origin was concentrated in open play (*n* = 69; 42.07%) and set pieces where the two penalty plays are included within the set-piece category for this descriptive breakdown (*n* = 49; 29.9%) followed by high-pressure recovery (*n* = 25; 15.24%) and counterattacks (*n* = 21; 12.80%). The shot-on-target rate was highest in high-pressure recovery (47.8%) and open play (44.8%), while the goal rate ranged from 11.7% (set pieces) to 19.0% (counterattacks) (Figure 6a). Regarding the type of combination, individual plays generated moderately longer progressions in set-piece and high-pressure recovery situations, while collective combinations tended to be shorter and more concentrated in open play (Figure 6b). 

## 4. Discussion

To the best of our knowledge, this is one of the first studies to conduct an integrated vector analysis of offensive performance in F5 by combining spatial, temporal, and contextual information on offensive plays in an official Paralympic competition. The results suggest that there are trends and patterns that may help explain spatiotemporal progression and decline in performance, although the multivariable predictors and temporal trend tests did not reach statistical significance, opponent differences in progression magnitude were significant (*p* = 0.019). Three findings emerge from the main results.

One could hypothesize that the magnitude of the progression vector is inversely related statistically to the probability of a shot on-target; it is the predictive factor most likely to be significant in the adjusted model and is often linked to players’ tendency to seek scoring opportunities. In the context of F5, this effect could be interpreted as a direct consequence of the progressive loss of useful acoustic information when the ball must travel long distances: greater distances imply more opposition, more competing sounds in the acoustic field (opponents shouting, “I’m coming!”, impacts against the boards, instructions from opposing coaches), and greater difficulty in the mental spatial representation of the player who must execute the actions, making spatial management a key factor that requires further research and depends on the player’s individual characteristics. In this regard, individualized analyses for each sport would allow for metrics tailored to the logic of each discipline and would improve analyses by focusing not only on the result but also on understanding the process [34,35].

This is consistent with other studies conducted with sighted athletes, which have confirmed that the ability to manage space and control in sports such as soccer are crucial indicators of success, especially since players’ movements have direct implications for the dynamics of the game [36]. Similarly, it has been noted that examining players’ posture in the analysis of offensive performance appears to be a determining factor in performance in highly tactically complex sports, such as team sports [37]. Other studies based on the analysis of shooting metrics in relation to body orientation in soccer have established that body orientation influences the quality of the shot [38]; therefore, estimating the probability of scoring a goal through a shot is suggested as an appropriate strategy. This is particularly relevant because, in F5, unlike conventional soccer, body orientation is guided not by visual information but by acoustic and proprioceptive cues.

This study found that each one-standard-deviation increase in vector length in the modeled subset (*n* = 148) reduces the OR that an attack will result in a shot on-target by 32% in high-level F5 competitions, when played on a 40 × 20 m field with four field players. Meanwhile, other studies analyzing professional soccer have found that only 1% of all offensive plays and nearly 10% of shots on goal result in a goal [39,40,41]. Thus, it can be concluded that finishing situations are complex due to the different metrics used to evaluate shooting opportunities in ecological models that analyze different spaces and game dynamics [21,42,43], so these performance metrics should be interpreted with caution. The reduction in goal probabilities could be due, according to the data from the present study, primarily to the fact that in F5, a greater distance covered by the same player during the attack leads to fewer shooting opportunities results that contrast with other studies that have evaluated the effectiveness of shooting on goal in F5, concluding that players who dribble the ball before shooting have a higher probability of scoring [16,18].

The area near the center goal saw zero goals on 13 attempts. In F5, this pattern may reflect both the team’s lateral specialization favoring shots from angles where players can orient their bodies using the kickboards as a tactile reference and the higher density of defensive acoustic information in central areas near the goal, where the opposing goalkeeper and defenders converge to create surprise, since the opposing goalkeeper does have visual information about the ball. These data do not align with other studies in elite futsal, where it has been reported that central zones are the most likely to favor shots on goal, accounting for 73% of all goals scored compared to the use of lateral zones [44]. Here, it should be noted that these orientations correspond not only to lateral specialization due to tactics and player positioning on the field but, especially, to the source of information used for decision-making [45] while the conventional futsal player relies on visual information, the F5 player relies on acoustic and proprioceptive information.

The study conducted by Valencia Sánchez et al. [46] reported that in the CONMEBOL Libertadores Futsal Cup (Uruguay 2021), most goals were scored in the central zone from distances of less than 6 m or more than 10 m, accounting for 74% of all goals scored. Meanwhile, in the present study, the spatial diversity of offensive effectiveness was marked and shifted toward the right side, with the area near the goal on the right side showing a conversion rate of 19%, compared to significantly lower rates in deep and central zones. In F5, this asymmetry reflects several non-mutually exclusive and specific factors, where it appears that the dominant functional laterality of the squad and individual preferences for body orientation based on acoustic cues may be due to years of adapted training [16]. This last observation is particularly relevant: unlike in conventional soccer, there is a hypothesis that the sidelines in F5 not only serve as boundaries of the playing field but could also function as active spatial references that players can incorporate into their mental map of the environment.

The contrast observed against Brazil, the current world champion against whom Argentina failed to score a single goal and managed only a 12.5% shot-on-target rate despite mounting 24 attacking plays with offensive runs that dispersed without converging toward the goal suggests at least two tactical hypotheses that are not mutually exclusive. On the one hand, this pattern appears to reflect Brazil’s defensive ability to thwart attacks at their source, which forced Argentina to initiate plays from more distant positions and with less direct paths toward the goal. On the other hand, it could be due to a strategic adaptation by the Argentine team itself toward a more conservative, low-risk style against the sport’s strongest rival, prioritizing ball possession over direct finishing. Both hypotheses have distinct practical implications: in the first case, to design solutions for build-up play and finishing against defenses that press the point of origin of the play; in the second, to plan offensive risk based on the opponent’s strength. These hypotheses should be tested in studies that incorporate defensive positional data which is currently excluded from this design to foster a deeper understanding of F5.

Finally, the data are consistent with a pattern of declining offensive performance during the game, with the goal rate dropping from 26.1% (minutes 6–10) to 6.7% (minutes 36–40, second half), representing a relative decrease of 74.3% in this study. These findings should be interpreted with caution and analyzed within the specific context of visually impaired players who rely on spatial cues derived from acoustic information. Therefore, other studies with visual data in conventional futsal confirm that there is an increase in intra-match offensive performance in relation to playing time, with the highest goal-scoring tendency occurring between the 10:01–15:00 and 30:01–35:00 periods [46], while, as the end of the match approaches, there is a higher prevalence of goals [47,48]. Further studies are needed to help understand the intra-match pattern in F5, as comparing these results with conventional futsal poses a methodological challenge in determining the factors that influence this decline in offensive performance due to the pace of play, team dynamics, positioning, etc.

### 4.1. Limitations

This study has several limitations that should be noted. The first limitation concerns the analysis of a single team in a single tournament, which makes it difficult to generalize the results. Another limitation stems from the failure to account for other tactical factors that may influence the results, such as playing style, the tactical level of opponents, individual player characteristics, and the opponent’s defensive positioning. The third limitation relates to the research design and the sample size analyzed, which limits the statistical power to identify the effects of the magnitude of the variables under analysis. A fourth limitation concerns the non-independence of observations: the offensive actions were nested within players (median 21 actions per player; range 2–37) and within matches, so the assumption of independent observations underlying the logistic model is unlikely to be fully satisfied and the standard errors may be optimistic. The appropriate remedy—a mixed-effects (multilevel) model with random intercepts for player and match—could not be reliably fitted given the small number of players (*n* = 8) and the low, uneven frequency of actions per player; the regression estimates should therefore be interpreted with this clustering in mind, and future, larger datasets should adopt a hierarchical specification. A fifth limitation relates to the origin coordinates, which were reconstructed from the original notation system and may contain parallax and spatial resolution errors. A sixth limitation stems from the failure to consider other performance indicators, such as physiological markers (e.g., heart rate), global positioning system (GPS) data, and cognitive load data (e.g., subjective cognitive fatigue reports or dual-task load), which could help elucidate the causal relationship between multimodal fatigue and decreased performance. A seventh limitation is linked to the observational design of the study, which prevents the establishment of causality.

### 4.2. Methodological Clarifications

Three methodological clarifications should be added to the above:

- First, the inter- and intra-observer reliability of the scoring system were not formally quantified; therefore, the reproducibility of the coding cannot be objectively guaranteed and should be established in future studies through the recording of a subsample (Cohen’s kappa, intraclass correlation coefficient, or standard error of measurement). Because every downstream analysis depends on the accuracy of the observational coding, we regard this as the principal methodological weakness of the study; at a minimum, a basic intra- and inter-observer estimate on a recoded subsample (e.g., Cohen’s κ or ICC for a random 15–20% of plays) should accompany future applications of this notation system.
- Second, as detailed in Section 2.3, the 16 plays excluded from the model for lack of origin coordinates were all goals; the multivariable estimates should therefore be interpreted as conditional on shots with a reconstructable origin vector.
- Third, the power analysis confirmed that the model was underpowered: for an OR of 0.68 per standard deviation and a prevalence of 35.8%, the estimated power with *n* = 148 was approximately 57%, and reaching 80% would have required around 240–260 plays according to the analytical approximation by Hsieh et al. [33]; this explains why the primary predictor barely reached significance (*p* = 0.051) and necessitates interpreting the results as exploratory and hypothesis-generating. The last limitation is associated with the failure to include defensive information from rival teams in the analysis, which may lead to difficulties in interpreting the results.

### 4.3. Future Research

About future research directions, the following suggestions are made. First, future studies should incorporate physiological markers and kinematic variables derived from GPS and inertial measurement units, as well as cognitive load markers, to help provide a clearer understanding of the decline in performance during offensive plays toward the end of matches. A second suggestion is to apply hierarchical Bayesian models to help integrate individual data. A third suggestion is to expand the analysis to include more competitions and levels, including youth tournaments and women’s blind soccer. Finally, studies are needed that use machine learning algorithms with SHapley Additive exPlanations (SHAP) interpretability to analyze performance on F5 [49].

### 4.4. Practical Applications

Based on the findings of this study, it is recommended to design training exercises focused on recovery under high pressure near the right side of the offensive zone and to strive to maintain the intensity of play in the final minutes of the game.

## 5. Conclusions

Spatial effectiveness was markedly asymmetrical: the area near the goal on the right side showed a 19% conversion rate, compared to 5–6% in deeper and central areas.

The results of this study suggest that the magnitude of the offensive progression vector and offensive effectiveness show an asymmetry toward the right side, as well as a pattern consistent with a decline over the course of the match toward its conclusion; these variables appear to influence the offensive performance of the Argentine men’s national blind football (F5) team in the 2022 Copa América tournament.

These findings provide one of the first integrated vector analyses of offensive performance in F5 and propose metrics applicable to coaching staff, the design of specific training programs, and the functional assessment of athletes with visual impairments; therefore, further studies are needed to analyze these metrics in different official competitions and to account for various competitive levels.

## Figures and Tables

**Table 1:** Descriptive statistics by opponent.

Opponent	*n*	Goals	On-Target	Goal Rate (%)	On-Target Rate (%)	Progression Mean (m)	Progression SD	Final-Distance Mean (m)	Final-Distance SD
Brazil	24	0	3	0.0	12.5	14.53	7.33	13.01	4.08
Chile	30	4	12	13.3	40.0	14.88	7.37	10.76	4.19
Colombia	36	8	22	22.2	61.1	15.63	7.05	11.02	4.06
Mexico	33	6	14	18.2	42.4	12.36	7.71	12.14	3.97
Peru	41	10	18	24.4	43.9	14.05	6.43	11.31	3.89

Note: *n*: number; %: percentage; m: meters; SD: standard deviation. Progression Mean and Progression SD are computed on plays with reconstructable origin coordinates (*n* = 148), which may differ from the column *n* for some opponents.

**Table 2:** Multivariable logistic regression for on-target outcome.

Predictor	β	SE	Odds Ratio	95% CI	*p*-Value
Intercept	−0.927	0.620	0.40	0.12–1.33	0.135
Progression vector (per +1 SD)	−0.387	0.199	0.68	0.46–1.00	0.051
Distance to goal (per +1 SD)	−0.481	0.309	0.62	0.34–1.13	0.120
Match minute (per +1 SD)	−0.273	0.185	0.76	0.53–1.09	0.140
Origin: Open play (vs. Counter-attack)	0.651	0.608	1.92	0.58–6.31	0.284
Origin: Set piece (vs. Counter-attack)	0.419	0.633	1.52	0.44–5.26	0.508
Origin: High-pressure recovery (vs. Counter-attack)	1.207	0.730	3.34	0.80–13.99	0.098
Combination: Individual (vs. Collective)	−0.404	0.408	0.67	0.30–1.48	0.322

Note: β: beta; SE: standard error; OR: odds ratio; CI: confidence interval; SD: standard deviation; vs.: versus; %: percentage.

**Table 3:** Outcome rates and progression by half.

Half	*n*	Goals	On-Target	Goal Rate (%)	On-Target Rate (%)	Progression Mean (m)	Progression SD
First half	89	18	41	20.2	46.1	14.80	6.7
Second half	75	10	28	13.3	37.3	13.67	7.62

Note: *n*: number; %: percentage; m: meters; SD: standard deviation.

**Table 4:** Spatial efficacy by pitch zone.

Depth Zone	Lateral Zone	*n*	Goals	On-Target	Conversion (%)	On-Target (%)
0–6 m (zone A)	Right wing	21	4	7	19.0	33.3
0–6 m (zone A)	Center	13	0	3	0.0	23.1
6–10 m (zone B)	Right wing	23	2	12	8.7	52.2
6–10 m (zone B)	Center	14	2	5	14.3	35.7
10–20 m (zone C)	Right wing	59	3	19	5.1	32.2
10–20 m (zone C)	Center	16	1	7	6.3	43.8

Note: *n*: number; %: percentage; m: meters. Depth zones: zone A (0–6 m), zone B (6–10 m), and zone C (10–20 m) correspond to the distance from the end point to the goal.

## Data Availability

The analyzed data is publicly available on the Kaggle repository (https://www.kaggle.com/datasets/agustingermanrojas/blind-football) (accessed on 15 June 2026).
